# An Event-Triggered Machine Learning Approach for Accelerometer-Based Fall Detection

**DOI:** 10.3390/s18010020

**Published:** 2017-12-22

**Authors:** I Putu Edy Suardiyana Putra, James Brusey, Elena Gaura, Rein Vesilo

**Affiliations:** 1 School of Engineering, Macquarie University, Sydney 2109, Australia; rein.vesilo@mq.edu.au; 2 Faculty of Engineering, Environment & Computing, Coventry University, CV1 5FB Coventry, UK; j.brusey@coventry.ac.uk (J.B.); e.gaura@coventry.ac.uk (E.G.)

**Keywords:** fall detection, accelerometer sensors, segmentation technique, fall stages, machine learning, computational cost

## Abstract

The fixed-size non-overlapping sliding window (FNSW) and fixed-size overlapping sliding window (FOSW) approaches are the most commonly used data-segmentation techniques in machine learning-based fall detection using accelerometer sensors. However, these techniques do not segment by fall stages (pre-impact, impact, and post-impact) and thus useful information is lost, which may reduce the detection rate of the classifier. Aligning the segment with the fall stage is difficult, as the segment size varies. We propose an event-triggered machine learning (EvenT-ML) approach that aligns each fall stage so that the characteristic features of the fall stages are more easily recognized. To evaluate our approach, two publicly accessible datasets were used. Classification and regression tree (CART), *k*-nearest neighbor (*k*-NN), logistic regression (LR), and the support vector machine (SVM) were used to train the classifiers. EvenT-ML gives classifier F-scores of 98% for a chest-worn sensor and 92% for a waist-worn sensor, and significantly reduces the computational cost compared with the FNSW- and FOSW-based approaches, with reductions of up to 8-fold and 78-fold, respectively. EvenT-ML achieves a significantly better F-score than existing fall detection approaches. These results indicate that aligning feature segments with fall stages significantly increases the detection rate and reduces the computational cost.

## 1. Introduction

Falls are a major health risk for the elderly. Age UK reported that falls are one of the leading causes of injury-related deaths and the main cause of disability and death for people aged over 65 in the UK [1]. The World Health Organization (WHO) also reports that falls are the second leading cause of injury-related deaths worldwide [2]. Falls can cause several types of injury, including fractures, open wounds, bruises, sprains, joint dislocations, brain injuries, or strained muscles [3]. Although fall detection systems are unable to prevent falls, they can reduce complications by ensuring that fall victims receive help quickly. Fall detection and alerting systems need to be automated because the fall victim may be unable to activate an alarm [4].

Generally speaking, accelerometer-based fall detection systems either use a threshold-based or a machine learning-based approach [5]. Threshold-based approaches detect falls by checking if the measured acceleration exceeds a predefined (and fixed) value [6,7,8]. Machine learning-based approaches use labeled data to train a *classifier* using supervised machine learning algorithms (e.g., the support vector machine (SVM), decision tree, and/or artificial neural networks) that can recognize the characteristic features of falls [9,10,11,12,13,14,15].

Threshold-based approaches are simple and have a low computational cost [5]. However, manually defining thresholds is difficult, as several activities of daily living (ADLs) can produce high acceleration (this can cause a high number of false alarms), while some falls may have a lower acceleration (this can cause falls to be left undetected) [15]. Furthermore, a comprehensive study from Bagala et al. [16] shows that the existing threshold-based approaches produce a high number of false alarms. One possible reason behind that high number of false alarms is that manually defined thresholds do not generalize well for unseen subjects [17]. Several studies used machine learning to construct a classifier that distinguishes falls from ADLs, so that the number of both false alarms and undetected falls can be reduced.

Machine learning-based approaches usually need a fixed-length overlapping sliding window (FOSW) [10,11,18] or a fixed-length non-overlapping sliding window (FNSW) [19] to split the data sequence into several segments. Then, features are extracted from each segment for training and testing of the classifier. For FNSW and FOSW, the feature extraction process is executed on all segments. This causes the computational cost of the system (which uses those sliding-window techniques) to increase. Also, this study shows that using an FOSW with a higher overlap can increase false alarms.

Putra et al. [14] proposed a new mechanism called the cascade-classifier approach (CCA), to reduce the computational cost of the system by extracting features only when the user’s body state is active. The body state is checked by using a 2-s FNSW and a threshold of 1.6 g (gravity). The state of the body is considered as active if the highest peak during the 2-s window is higher than 1.6 g, and this peak is used as an indicator of the impact stage. When the body state is active, another window with a length of 12 s is fitted to the acceleration signal for feature extraction, where 1 s before the highest peak is considered as the pre-impact stage and 11 s after the highest peak is considered to include the impact and post-impact stages.

CCA also shows that extracting features based on fall stages (pre-impact, impact, and post-impact) yields improved detection rates. However, fitting the fall stages to a segment is a non-trivial task since the estimation of the beginning and the end of each stage in a segment is unclear. A study from Abbate et al. [20] used acceleration peaks to identify the start of the impact stage. Nevertheless, during a fall (in the impact stage), multiple high peaks can be produced, which can confuse the segmentation process. Moreover, based on Jamsa et al. [21], peaks can also occur during the pre-impact stage as a result of protective actions. The presence of multiple peaks (that we call the multi-peak problem) makes the estimation of the impact stage even harder as it can mislead the alignment of the beginning of that stage. Although the multi-peak problem is important for the data segmentation process, it has not been considered in previous studies [14,18].

The main contribution of this paper is to provide a solution to the multi-peak problem, with the aim of correctly aligning fall stages to a segment so that the detection rate can be significantly improved. This paper develops a novel approach called event-triggered machine learning (EvenT-ML) with multi-peak detection for fall detection using wearable sensors. This approach consists of:

- The use of a finite state machine to align fall stages (pre-impact, impact, and post-impact) with a segment. These fall stages are used as a basis for feature extraction.
- A mechanism to resolve the ambiguity caused by multiple peaks so that the alignment of each stage of the fall can be correctly estimated.

To demonstrate the performance of our approach , FNSW- and FOSW-based machine learning approaches were implemented for comparison. Our experimental results show that EvenT-ML can achieve a significantly better F-score than FNSW or FOSW. As an additional advantage, EvenT-ML has a significantly lower computational cost than the FNSW- and FOSW-based machine learning approaches. For this application, reducing the computational cost of the algorithms running on the wearable device is important as it reduces power consumption and thus extends battery life [22]. In addition, we compared EvenT-ML to two existing fall detection approaches: a threshold-based approach (IMPACT+POSTURE) from Kangas et al. [7] and a fall stages-based machine learning approach from Putra et al. [14]. Compared to those techniques, our approach is able to achieve a significantly better F-score.

The rest of the paper is organized as follows. Section 2 introduces related work. Our event-triggered machine learning approach is explained in Section 3. Section 4 and Section 5 provide our experimental evaluation and parameter justification for EvenT-ML, respectively. Section 6 provides results, while Section 7 gives conclusions as well as prospective future work.

## 2. Related Work

Threshold-based approaches are commonly used in existing fall detection systems as they have a low computational cost [5]. These approaches use manually pre-defined thresholds to classify falls [6,7,8]. Manually defining thresholds to accurately classify falls is not a trivial task. Bourke et al. [23] and Kangas et al. [24] proposed box-plot analysis to identify threshold values that can distinguish falls effectively. However, Vallejo et al. [15] found that using only box plots to define thresholds is ineffective since fall and non-fall activities overlap in terms of acceleration. This overlap causes threshold-based approaches to produce a high number of false alarms.

To reduce the number of false alarms while maintaining a high detection rate, several studies used machine learning as an alternative [10,11,15,18,19]. Vallejo et al. [15] developed an artificial neural-network-based approach for fall detection using FNSW with a 10-sample length. Based on their experiments using a laboratory-based dataset, their approach produced 6 false alarms and missed 6 fall events (false negatives) from 381 falls and 429 non-fall activities. Their approach was also able to generate zero false alarms in a real-life case study of 12 h.

Erdogan and Bilgin [19] developed an FNSW-based machine learning approach with k-nearest neighbor (k-NN). They used an accelerometer strapped to the subject’s waist. The length and the offset of the window are not specified. Their approach achieves 100% recall and 85% precision. There is no further information regarding the data collection procedure for this study.

Dinh and Struck [11] tried to combine a fuzzy-logic inference system and an artificial neural network to classify falls based on acceleration data from five subjects. By using an FOSW with a length of 5 s and offset of 0.5 s on a laboratory-based fall dataset, their approach was able to achieve up to 90.3% precision, 96% sensitivity, and 99.8% specificity.

A study conducted by Diep et al. [10] used a Wii remote controller as a low-cost fall detector with a support vector machine (SVM)-based classifier, using acceleration data from 12 subjects in a laboratory environment. Their approach used FOSW with a length of 1.8 s and an 0.6 s overlap, and was able to reach 91.9% precision and 94.4% recall.

Although the different studies above used different sensor types and a different number of activity types, the general pattern is that precision tends to be lower than recall. In real-world deployment, a fall detection system needs to balance precision and recall performance. Lower recall means that the system may miss an important event but low precision means a high number of false alarms, which may cause users to reject the system [25].

Ojetola [18] provides a method that uses FOSW to segment the data stream. He implemented a 12-s sliding window with an overlap of N−1, where *N* is the total number of samples in the window, and used a decision tree as the classifier. To improve the classifier’s detection rate, fall stages (pre-impact, impact, and post-impact) were aligned to each segment as a basis for feature extraction. During the pre-impact stage, acceleration drops below 1 g since the subject is (briefly) in free fall after losing balance. The impact stage of the fall coincides with one or more high peaks in the accelerometer measurements. The highest peak during the impact stage is generally taken to be the moment when the body hits the floor. The post-impact stage is characterized by inactivity corresponding to reduced variation in the accelerometer reading. For an experimental, laboratory-based dataset, this approach achieves a precision, recall, and F-score of 93.5%, 94%, and 93.2%, respectively. Notably, both precision and recall are high.

Although Ojetola improved the detection rate, our prior work [14] showed that the method has a high computational cost because it has to extract features for every possible segment produced by the sliding window. A cascade-classifier approach (CCA) was developed to reduce this cost, and it also improved the classification performance. It is acknowledged that complex features increase the system’s computational cost [22]. Therefore, CCA reduces the computational cost of the system by extracting features only when energetic activity is detected. Thus, CCA has a lower computational cost than FNSW and FOSW. Although CCA is able to reduce the computational cost, it does not correctly identify the temporal position of stages when multiple peaks appear.

In this study, we propose an improvement of CCA. We use the concept of fall stages from Ojetola’s study [18] as a basis for feature extraction and a state machine to fit the fall stages to the segment. Table 1 shows improvements in functionality of EvenT-ML compared to Ojetola’s method and CCA.

## 3. An Event-Triggered Machine Learning (EvenT-ML) Approach

**Figure 1:**
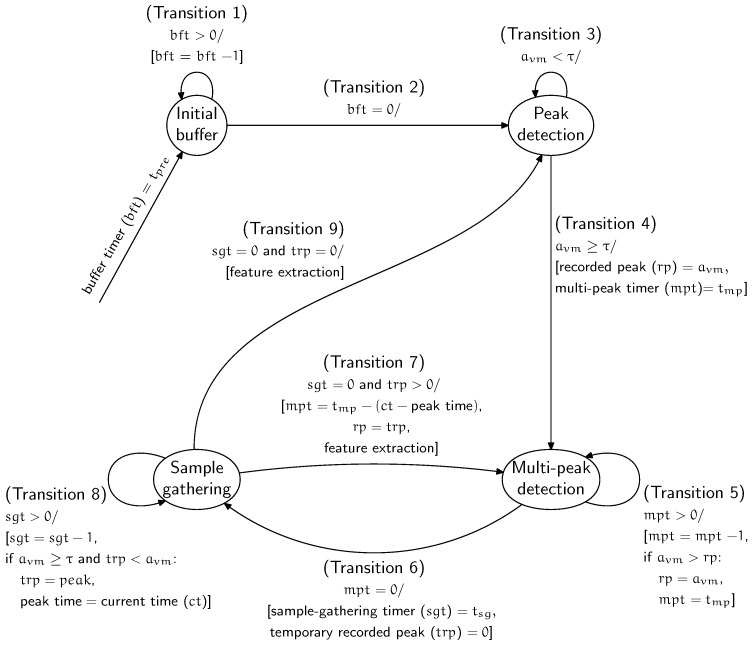
State machine for EvenT-ML.

This section describes the procedure of EvenT-ML for segmenting the data based on fall stages (pre-impact, impact, and post-impact). EvenT-ML’s operation can be described as a finite state machine (FSM) to ensure that the detection algorithm can be executed on-line with minimal memory requirements. EvenT-ML uses four states: **Initial buffer**, **Peak detection**, **Multi-peak detection**, and **Sample gathering**. Figure 1 shows the EvenT-ML state machine.

**Initial buffer**: When the system is started, this state is executed once. This state collects samples as an initial buffer. The idea of this buffer is to provide enough samples for the pre-impact stage. This state uses a timer called buffer timer (*bft*) with a length of $t_{pre}$.

**Peak detection**: This state looks for peaks in the acceleration vector magnitude ($a_{vm}$). A peak is an $a_{vm}$ that is higher than a threshold (τ). If a peak occurs, it is assumed that the subject is active and the state of the system is changed to **Multi-peak detection**.

**Multi-peak detection**: During a fall, several acceleration peaks can be produced. EvenT-ML assumes that, if a fall has occurred, then the highest peak corresponds to the moment when the body hits the ground (impact stage) [18]. EvenT-ML identifies the alignment of the impact stage by finding the highest peak during a particular length of time ($t_{mp}$). If there is another peak higher than the *recorded peak*, this is taken as the new highest peak and the counter (*mpt*) of this state is reset. This counter ensures that the length of the impact stage is equal to $t_{mp}$. The pre-impact stage is defined as all samples before the *recorded peak* with a length of $t_{pre}$. This state differentiates EvenT-ML from CCA [14], where CCA does not have a mechanism to detect multiple peaks. This mechanism is important to estimate the beginning of the impact stage.

**Sample gathering**: After detecting the highest peak, further samples during a certain amount of time ($t_{sg}$) are collected so that a complete fall segment (including pre-impact, impact, and post-impact stages) can be captured. If there is an $a_{vm}$ higher than τ, that $a_{vm}$ value is stored as a *temporary recorded peak* and the *current time*(*ct*) is recorded. Both the *temporary recorded peak* value and its time are updated if another higher peak occurs. The *temporary recorded peak* concept is important in order to avoid missing any peaks, as those peaks could be an indicator of a fall. When the counter (*sgt*) for this state has ended, feature extraction is executed. After performing feature extraction, if the value of the temporary recorded peak is equal to 0, the state is changed to **Peak detection**. Otherwise, the state is changed to **Multi-peak detection** and the *temporary recorded peak* is set as a new *recorded peak*. Then, the *mp timer* is set to $t_{mp}-\left(\mathrm{ct}-\mathrm{peaktime}\right)$.

The state machine above produces a segment where: all samples during $t_{pre}$ (in seconds) before the highest peak are considered as the pre-impact stage; all samples during $t_{mp}$ (in seconds) starting from the highest peak are considered as the impact stage; and all samples during $t_{sg}$ (in seconds) after the impact stage are considered as the post-impact stage. Section 5 provides a parameter selection for $t_{pre},\;t_{mp},\;t_{sg}\;\mathrm{and}\;\tau$.

Several features are needed during the training and testing processes of the classifier. The following features were used in this study:

1. Minimum, maximum, and average acceleration vector magnitudes [14,18,20].
2. Velocity [6,14,18].
3. Energy expenditure [18,26].
4. Variance of the acceleration vector magnitude [14,18,27].
5. Root mean square (RMS) of the acceleration vector magnitude [6,14,28].
6. Acceleration exponential moving average (EMA) [14,18,27].
7. Signal magnitude area (SMA) [14,18,29].

The features above are extracted from the pre-impact, impact, and post-impact stages (27 features in total).

## 4. Experimental Evaluation

### 4.1. Falls and Activities of Daily Living (ADLs) Dataset

Since obtaining real fall data from the elderly is a challenging task, and using the elderly to stage falls is inappropriate as it might harm their well-being, datasets from Ojetola et al. [30] (Cogent) and Sucerquia et al. [31] (SisFall) were instead used in this study (The Cogent dataset can be downloaded at: http://skuld.cs.umass.edu/traces/mmsys/2015/paper-15/. The SisFall dataset can be downloaded at: http://sistemic.udea.edu.co/en/investigacion/proyectos/english-falls/). Ethical approval for the experimental procedure for the Cogent and SisFall datasets was obtained from Coventry University and the Universidad de Antioquia UDEA (Medellín, Colombia), respectively. Although Noury et al. [25] and Abbate et al. [20] proposed more activities in their datasets, those datasets are not publicly accessible. Using a publicly accessible dataset is important as it can give a fair comparison between techniques [32].

The Cogent and SisFall datasets were collected from young and middle-aged healthy subjects. Using data from young and middle-aged healthy subjects to evaluate a fall detection approach is still debatable. Jamsa et al. [21] and Kangas et al. [33] showed that real falls of the elderly had a similar pattern to laboratory-based falls using young and middle-aged subjects. Jamsa et al.’s study also confirms that real forward, sideways, and backward falls have pre-impact and impact stages. Thus, the results of our study might indicate the performance of our approach with real fall events of the elderly.

#### 4.1.1. Cogent Dataset

The data were gathered from 46 healthy subjects (9 females and 37 males, age 23.5 ± 5.5 years, height 172.7 ± 7.7 cm, and weight 69.7 ± 12.8 kg). Each subject was required to stage 14 falls (including 6 forward, 4 backward, and 4 lateral falls) and several ADLs for 23 min on average. In total, this dataset has 644 fall and 1196 ADL events, representing a larger dataset than is found in several similar studies, such as those of Noury et al. [25] (600 data points for both falls and ADLs) and Abbate et al. [20] (86 fall-like events with 44 falls included). Also, Ojetola et al.’s dataset has near-fall scenarios where those scenarios can cause false alarms in the real-world case [8]. Table 2 shows the types of falls and ADLs (followed by their number of instances) of this dataset. More detailed information regarding the protocol can be found in [30]. Sensors were strapped to the chest and thigh of each subject. Some subjects also had a sensor strapped to their waist. Shimmer sensors [34] with a sampling rate of 100 Hz were used for data collection. The Shimmer consists of a 3D accelerometer, 3D gyroscope, Bluetooth radio, and an MSP430F1611 microcontroller. The data were transferred to a personal computer (PC) using Bluetooth and they were manually annotated with LabView.

#### 4.1.2. SisFall Dataset

The data are divided into two groups: those of young adults and elderly people. For this study, we only used data from the young-adult group because the elderly group does not involve any fall activities. This young-adult group consists of 10 males and 11 females (age 25.0 ± 8.6 years, height 165.7 ± 9.3 cm, and weight 57.7 ± 15.5 kg). The number of subjects used in this study is lower than in the published dataset, as some subjects were removed due to incomplete samples. Each subject was asked to perform 15 types of falls five times, where each fall takes three seconds. Moreover, to gather ADL data, each subject was asked to perform 19 types of ADLs. Thus, this dataset has 1575 fall and 1659 ADL data points. Table 3 shows the types of falls and ADLs (followed by their number of instances) of this dataset. More detailed information about the protocol used for this dataset can be found in Sucerquia et al. [31].

To collect the data, a custom-made device that consists of a Kinetis MKL25Z128VLK4 microcontroller (NPX, Austin, TX, USA), an Analog Devices (Norwood, MA, USA) ADXL345 accelerometer, a Freescale MMA8451Q accelerometer, an ITG3200 gyroscope, an SD card for recording, and a 1000 mA/h generic battery was used. The device was placed on the subjects’ waists. For the study in this paper, only the data gathered from the ADXL345 accelerometer are used.

### 4.2. Experimental Setup and Evaluation

This study implemented a binary classification, which means all different types of falls are considered as being falls, while other activities are considered as being non-falls. For training and testing the classifier, a classification and regression tree (CART), *k*-nearest neighbor (*k*-NN), logistic regression (LR), and support vector machine (SVM) from the Scikit-learn library [35] were used. Leave-one-subject-out cross-validation is used in this experiment as the classifier evaluation method. This method is used because it is able to determine the ability of the classifier to generalize to unseen subjects and helps to identify the classifier’s variability from subject to subject [13,36].

As the number of fall data is very small compared to ADLs (data imbalance), accuracy cannot be used to measure the classifier’s performance because it overvalues the always-negative classifier [37]. Thus, precision, recall, and F-score were used as the performance measurement metrics in this study. The precision, recall, and F-score (harmonic mean of precision and recall) are given by

(1) $$\begin{array}{ccc} \mathrm{Precision} & = & \frac{\mathrm{TP}}{\mathrm{TP}+\mathrm{FP}} \end{array}$$

(2) $$\begin{array}{ccc} \mathrm{Recall} & = & \frac{\mathrm{TP}}{\mathrm{TP}+\mathrm{FN}} \end{array}$$

(3) $$\begin{array}{ccc} F-\mathrm{score} & = & \frac{2\mathrm{TP}}{2\mathrm{TP}+\mathrm{FP}+\mathrm{FN}} \end{array}$$

where TP, FP, and FN are the numbers of true positives, false positives (or false alarms), and false negatives (or undetected falls), respectively.

Consider a data sequence that has been annotated as a particular activity during data collection. When that sequence is segmented for online processing by a technique such as FNSW, FOSW, or EvenT-ML, several segments will usually be produced. Since each segment is classified separately, the segment classifications produced may be different. To obtain a single classification result for a data sequence of an activity, the following rules are implemented:

- A data sequence is detected as an FP if this data sequence is annotated as a non-fall activity and at least one segment is detected as a fall.
- A data sequence is detected as a TP if this data sequence is annotated as a fall activity and at least one segment is detected as a fall.
- A data sequence is detected as an FN if this data sequence is annotated as a fall activity and no segment is detected as a fall.

To evaluate activities that are often misclassified as falls and falls that are often misclassified as non-falls, the false positive ratio (FPR), and false negative ratio (FNR) of each activity and fall are calculated by using the following formulas:

(4) $$FPR_{i}=\frac{FP_{i}}{p},$$

(5) $$FNR_{i}=\frac{FN_{i}}{q},$$

where $FP_{i}$, $FN_{i}$, *p*, *q*, and *i* are the number of times that the ith type of activity is misclassified as a fall, the number of times that the ith type of fall is misclassified as a non-fall, the total number of samples of ith type of activity, the total number of samples of ith type of fall, and the type of fall or activity, respectively. An activity/fall with a higher FPR/FNR means that particular activity/fall is harder to detect.

For this study, we focused on reducing the computational cost of the data pre-processing (data segmentation and feature extraction). Thus, we use a modified model of total energy consumption (*E*) from Dunkels et al. [38],

(6) $$\frac{E}{V}=I_{m}t_{m},$$

where *V*, $I_{m}$ and $t_{m}$ are the the supply voltage, current draw, and the running time of the microprocessor in normal mode, respectively.

It is assumed that two segments are implemented on the same sensor device, so that the current draw and the supply voltage are the same for each segment ($I_{m_{1}}=I_{m_{2}}$ and $V_{1}=V_{2}$). This shows that energy is proportional to running time: E∝t. Thus, running time was used as a metric to measure the computational cost of the data pre-processing in this study.

The significance of the improvement (for both detection rate and computational cost) was evaluated using a Wilcoxon signed-rank test with a significance level of 0.05 (α=0.05). This method was chosen because the distribution of the precision, recall, F-score, and computational cost values are not normal (based on Shapiro–Wilk normality test).

## 5. Parameter Selection for EvenT-ML

From Section 3, it can be seen that Event-ML has some parameters to define: the pre-impact ($t_{pre}$), impact ($t_{mp}$), and post-impact ($t_{sg}$) window sizes and a threshold (τ). This section discusses a selection process of these parameters, to get the highest F-score for Event-ML.

Two publicly-accessible datasets were available for this study: Cogent [30] and SisFall [31]. The Cogent dataset has more variations in terms of the length of both falls and ADLs than the SisFall dataset. This is because the SisFall dataset has a uniform length (15 s) for its fall data. This length can predictably give a better F-score for the classifier (see [39] for an analysis of window size on the SisFall dataset). However, the length of human activities is unpredictable in real cases. Thus, the result from the SisFall dataset might not be able to represent the performance of the classifier in the real cases. This means that the SisFall dataset might not be appropriate to define the window size. Thus, this section uses the Cogent dataset to select the parameter values of EvenT-ML.

### 5.1. Choice of the Threshold (τ)

The aim of the threshold (τ) is to ensure that only energetic activities are forwarded for feature extraction. Several existing studies propose some thresholds for detecting energetic activities: 1.6 g [14,18,20], 1.8 g [22], and 1.9 g [40]. Based on the Cogent dataset, the minimum of the impact-peak value (the value of the highest peak that is produced when the body of the subject hits the ground) of fall data across the Cogent dataset was found to be 1.8 g. In contrast, the threshold from Chen et al. [40] (1.9 g) is higher and causes some falls that will not be captured for feature extraction. Thus, thresholds of 1.6 g [13,14,20] and 1.8 g [22] are considered in this study.

### 5.2. Choice of the Size for the Fall Stages

Because the dataset does not provide annotation or video to distinguish between the pre-impact stage and the impact stage, it is hard to estimate the precise lengths of those stages. A study from Ojetola [13] shows that using a 1-s window for the pre-impact stage on the Cogent dataset can give a relatively good F-score. Thus, we used 1 s for $t_{pre}$ as proposed by Ojetola’s study.

Because the pre-impact stage is assumed to be 1 s and most falls (pre-impact and impact stages) occur in 2 s or more, the minimum impact-stage time is 1 s. Ojetola’s study [18] suggested 6 s as the impact-stage time to get a better F-score. Based on this, we considered the use of a 1–6 s of window for the impact stage.

As some of the post-impact stages from the dataset were supervised (for example, the subjects were asked to remain lying down for 10 s after falling), the length of the post-impact stage of this dataset was less natural because in the real case the length of this stage is unpredictable (the victim might lie on the floor longer if he/she faints). Thus, to get the post-impact stage size that could give the best F-score, a range of 1–6 s [41] for the post-impact stage was considered.

### 5.3. Parameter Selection Results

To select the values of the parameters of EvenT-ML, in order to get the maximum F-score, an experiment was done using some machine learning algorithms: CART, *k*-NN, LR, and SVM on the Cogent dataset. The following parameter values were considered for tuning the *k*-NN, LR, and SVM algorithms:

- *k*-NN with k=1,2, and 3. Euclidean distance and a uniform weight for all features are applied to the *k*-NN in this study;
- LR with an inverse regularization strength (*C*) of $10^{8},10^{9},$ and $10^{10}$;
- SVM with linear, polynomial, and radial basis function (RBF) kernels.

To choose the best value of *k* for *k*-NN, *C* for LR (where these parameters are called hyper-parameters), and the best kernel for SVM, parameter values above were tested, and the ones that can give the best F-score were selected.

**Figure 2:**
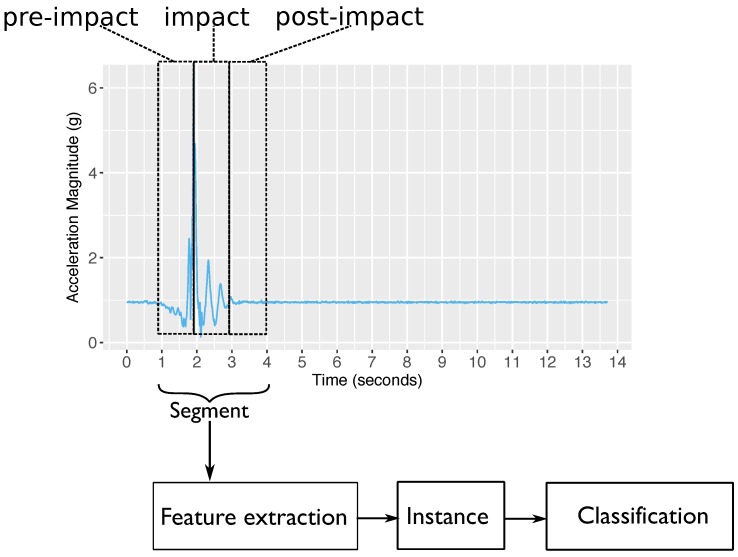
A segment produced by EvenT-ML.

The experiment results show that increasing either the impact or post-impact window size does not necessarily increase the F-score. The best window sizes for both the impact ($t_{mp}$) and post-impact ($t_{sg}$) stages are 1 s. In terms of F-score, using τ=1.8 g (when impact and post-impact window sizes are 1 s) gives a significantly better result than using τ=1.6 g (*p*-value = 0.02 ). Based on these findings, Figure 2 shows an example of a fall segmentation produced by EvenT-ML. 

With regard to the machine learning parameters, Table 4 shows F-scores of *k*-NN-, LR-, and SVM-based classifiers when $t_{mp}=1$ s, $t_{sg}=1$ s, and τ=1.8 g, and the following parameter values for the machine learning algorithms are chosen for this study, as they can give the best F-score:

- k=3 for *k*-NN,
- $C=10^{9}$ for LR,
- Linear kernel for SVM.

In fact, for the LR-based classifier, there is not a significant difference between using $C=10^{8}$, $C=10^{9}$, or $C=10^{10}$ (*p*-values ≥0.4). These parameters are used by Event-ML for a comparison with the sliding-window-based (FNSW and FOSW) approaches, CCA [14], and IMPACT+POSTURE [24] in the next sections.

## 6. Results , Analysis, and Discussion

### 6.1. Machine Learning-Based Fall Detection Approach Performance Analysis

This subsection aims to show the effectiveness of EvenT-ML in increasing the classifier’s performance (in terms of precision, recall, and F-score) and its ability to reduce the computational cost of the sliding-window-based (FNSW and FOSW) approaches. To compare EvenT-ML with FNSW- and FOSW-based approaches using the Cogent dataset, the data from the chest sensor were used because the chest is widely considered to be the best placement to achieve a high detection rate for fall detection [18,28]. For the SisFall dataset, the data from the waist sensor were used because this dataset only has one sensor placement. Table 5 shows the precision, recall, and F-score of EvenT-ML and sliding window-based (FNSW and FOSW) approaches on different machine learning algorithms using the Cogent and SisFall datasets.

Using EvenT-ML significantly improves precision when compared with the FNSW or FOSW approaches on both the Cogent and SisFall datasets regardless of the machine learning algorithm (*p*-values ≤0.01). By examining the precision values, it can be seen that EvenT-ML has a lower number of false alarms than FNSW and FOSW. In fact, using FOSW produces more false alarms than using either EvenT-ML or FNSW.

In terms of recall, EvenT-ML improves on both the FNSW and FOSW approaches when LR or SVM is used on the Cogent dataset. However, EvenT-ML achieves lower recalls than FNSW- and FOSW-based approaches when the SisFall dataset is used.

Generally, our approach achieves the best F-scores compared to both FNSW- and FOSW-based approaches on both datasets. This means that our approach is able to reduce the number of false alarms while not increasing the number of false negatives. Based on the Wilcoxon signed-rank test, EvenT-ML can significantly outperform both FNSW- and FOSW-based approaches in terms of F-score (all *p*-values $\;\leq 4.9\times 10^{-5}$). These results show that correctly segmenting the data based on fall stages and extracting features from those stages can improve the overall detection rate (in terms of F-score). We also found that EvenT-ML achieved better precision, recall, and F-score when the Cogent dataset was used. This can be an indicator that the chest placement (the Cogent dataset) is better than the waist placement (the SisFall dataset).

Table 6 shows the average numbers of segments that are produced by each approach and the total computational cost of the feature extraction for each subject. Based on the test results, EvenT-ML has significantly fewer segments over which it runs feature extraction than FNWS and FOSW (all *p*-values $\;\leq 4.3\times 10^{-5}$) on both datasets. In terms of total computational cost, EvenT-ML is able to achieve an up to 78-fold run-time reduction compared to FOSW and on average an 8-fold reduction compared to FNSW on the Cogent dataset. For the SisFall dataset, EvenT-ML involves a significantly lower computational cost than both FNSW (2-fold) and FOSW (up to 20-fold). Based on the Wilcoxon signed-rank test, EvenT-ML achieves a significantly lower computational cost than both FNSW and FOSW, with all *p*-values $\;\leq 9.5\times 10^{-13}$.

EvenT-ML, therefore, consumes significantly less energy than FNSW and FOSW, since energy is proportional to running time. The computational cost of EvenT-ML can potentially be further reduced by lowering the sampling rate of the signal. This will be investigated in the future.

### 6.2. A Comparison Between EvenT-ML and CCA

We previously developed CCA [14] with the aim of improving the detection rate of the fall-stages-based machine-learning approach from Ojetola [18] while reducing its computational cost. Although CCA is able to reduce the computational cost of Ojetola’s technique, CCA is unable to handle the multi-peak issue.

Table 5 shows the precision, recall, and F-score of the CCA-based machine learning approaches on the Cogent and SisFall datasets. Compared to FNSW and FOSW, CCA is able to achieve a higher precision in most of the cases, except when the Cogent dataset and LR or SVM algorithm are used. On the other hand, CCA achieves a lower recall than FNSW- and FOSW-based approaches in most of the cases, except when the Cogent dataset and SVM algorithm are used. In terms of the F-score, CCA outperforms both the FNSW- and FOSW-based approaches in most of the cases, except when the Cogent dataset and LR or SVM are used.

Compared to CCA, EvenT-ML is able to achieve improved precision and recall regardless of the machine learning algorithm. In terms of F-score, EvenT-ML can achieve significantly better performance than CCA on both the Cogent and SisFall datasets (*p*-values $\;\leq 2.4\times 10^{-4}$). These results show that extracting features based on the fall stages alone cannot significantly increase the classifier’s F-score. However, extracting features based on the fall stages and handling multiple acceleration peaks leads to a significant improvement in the classification performance (in terms of precision, recall, and F-score).

### 6.3. A Comparison between the EvenT-ML and IMPACT+POSTURE

Although a threshold is applied with EvenT-ML, this approach is different from a threshold-based approach. The main difference is that the classifier of EvenT-ML is built by a machine learning algorithm, while the classifier of the threshold-based approach is built using manually-defined thresholds. In this section we analyze the IMPACT+POSTURE algorithm (one of the threshold-based algorithms) that was proposed by Kangas et al. [7] and compare this technique to our approach.

#### 6.3.1. IMPACT+POSTURE

The IMPACT+POSTURE algorithm uses five parameters as thresholds:

- Total sum vector $SV_{tot}$;
- Dynamic sum vector $SV_{D}$;
- The difference between the maximum and minimum of the total sum vector $SV_{maxmin}$;
- Vertical acceleration $Z_{2}$; and
- Posture.

A more detailed explanation of the parameters above can be found in Kangas et al. [7]. This algorithm detects falls by checking the values of $SV_{tot}$, $SV_{D}$, $SV_{maxmin}$, and $Z_{2}$ first, then the posture is also checked if one of the first four parameters exceeds its pre-defined threshold.

The threshold-based approach is less general than a machine learning-based approach since the values of thresholds depend on the way they are defined and the way data are collected (e.g., the type of the accelerometer and the subject’s body profile). We followed the approach from Kangas et al. [24] to generate threshold values for IMPACT+POSTURE’s parameters ($SV_{tot}$, $SV_{D}$, $SV_{tot}$, $SV_{maxmin}$, and $Z_{2}$). For the posture, we used the threshold of 0.5*g* from Kangas et al. [7].

#### 6.3.2. Performance Comparison

To evaluate IMPACT+POSTURE’s performance, we found the precision, recall, and F-score of the IMPACT+POSTURE algorithm and compared these values with our approach using the Cogent and SisFall datasets, as shown in Table 7. IMPACT+POSTURE is evaluated with leave-one-subject-out cross-validation where the thresholds are adjusted using N-1 subjects (N is the total number of subjects) and one subject is used as a test case, (this process is repeated until all subjects have been a test case once).

For the Cogent dataset, IMPACT+POSTURE is able to achieve a significantly better F-score than the FNSW- and FOSW-based machine learning approaches when *k*-NN or CART is used to train the classifier (*p*-values $\leq 4.4\times 10^{-}5$). Compared to FNSW- and FOSW-based machine learning approaches using the SisFall dataset, IMPACT+POSTURE is able to achieve a significantly better F-score in most of the cases (*p*-values ≤0.02) except when the machine learning-based approach uses an FNSW and *k*-NN (*p*-value = 0.3) to train and test the classifier. See Table 5 for the results of FNSW- and FOSW-based machine learning approaches. The results above show that a simple threshold-based approach (IMPACT+POSTURE) is able to significantly outperform the machine learning based approaches in some cases, when the fall stages are not correctly aligned with the data segment.

On the other hand, our experiment showed that the IMPACT+POSTURE algorithm achieves lower precision and recall than EvenT-ML when the Cogent dataset is used. For the SisFall dataset, EvenT-ML achieves less recall but significantly higher precision than IMPACT+POSTURE (*p*-values $\;\leq 4.3\times 10^{-5}$). In general, EvenT-ML achieves a significantly better F-score than IMPACT+POSTURE regardless of the machine learning algorithm on both the Cogent and SisFall datasets, with *p*-values ≤0.03. See Table 5 for the results of EvenT-ML approach. These results show that EvenT-ML is able to reduce the number of false alarms, while still maintaining a recall comparable to IMPACT+POSTURE.

### 6.4. False Negatives and False Positives Analysis

**Figure 3:**
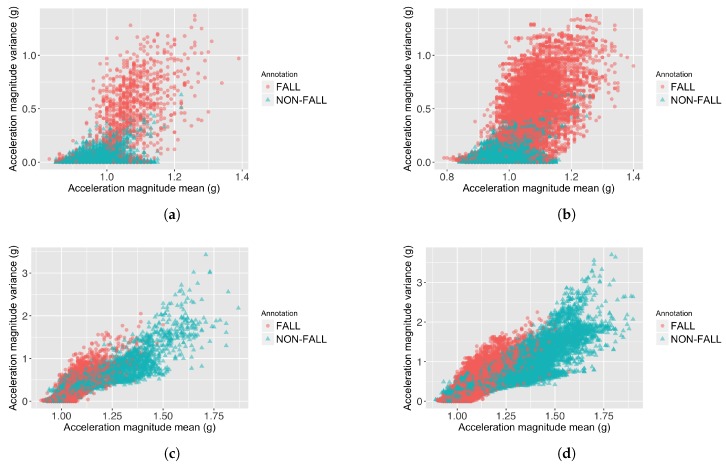
Data overlaps caused by increasing the window overlap on FOSW. (**a**) Feature value distribution of 25%-FOSW on the Cogent dataset; (**b**) Feature value distribution of 90%-FOSW on the Cogent dataset; (**c**) Feature value distribution of 25%-FOSW on the SisFall dataset; (**d**) Feature value distribution of 90%-FOSW on the SisFall dataset.

This study uses two large publicly accessible datasets with a relatively wide range of activities and falls, so that all techniques are fairly compared. An important finding of the classifier performance (shown in Table 5) is that increasing the window overlap in continuous feature extraction FOSW can cause the precision to reduce, because the number of data overlaps between fall and non-fall samples is increased on both datasets. This data overlap phenomena is shown in Figure 3. This means that using an FOSW with a high overlap (e.g., micro-annotation-based machine learning approach from Ojetola et al. [18]) is not effective in increasing the classifier’s overall performance (F-score).

The near-fall is the activity that produces the highest number of false alarms (see Formula 4 to calculate the false positive ratio), while fall-to-the-right-side becomes the hardest fall to detect (see Formula 5 to calculate the false negative ratio) from the Cogent dataset for most of the classifiers. For the SisFall dataset, the hardest fall to detect is fall-forward while sitting (F13). With regard to false alarm, different classifiers find different types of activities hard to detect.

These results imply that near-fall shares similar features with fall. This is because near-fall accompanies an abrupt movement, where this abrupt movement is similar to a fall [8]. Fall-to-the-right-side and fall-forward while sitting (F13) share similar features with non-fall activities. Due to data limitations (for example a lack of videos of all of the subjects performing a fall, and data on the subject’s preferred hand or any preventive actions when the subject senses he/she is about to fall) and a complex interaction between fall dynamics and machine learning algorithm used, a further investigation regarding the fall that produces the highest false negative cannot be thoroughly completed.

This is a limitation of this study. Also, because this study implements a binary classification technique, the classifier recognizes all falls as only one class, regardless of their type. This condition makes classifying falls based on their type another limitation of this study. Type-based classification can be done by using a multi-class classification technique [42], and should be considered for future work.

### 6.5. Energy Consumption Discussion

Increasing the window overlap can also significantly increase the computational cost of the system, as the number of feature extraction processes increases. Having a high computational cost system on a small wearable device is a disadvantage, as it causes the microprocessor to work longer. This increases the energy consumption of the device (E∝t), which thus drains the battery quickly.

Table 6 shows that EvenT-ML has significantly fewer segments than FNSW and FOSW, which means EvenT-ML executes fewer feature extractions than approaches using sliding window techniques. This means that EvenT-ML has a lower computational cost than FNSW and FOSW, and thus EvenT-ML is likely to consume less energy than FNSW- and FOSW-based approaches. Note that the degree of energy reduction in a real device might be different from the results presented in this study, because there are other processes that might increase the energy consumption of the real wearable device.

## 7. Conclusions and Future Work

This paper describes a novel technique, called the event-triggered machine learning (EvenT-ML) approach, for human fall detection. This algorithm provides a mechanism to prevent feature extraction from being executed all the time by extracting features only when the subject is in the active state. It also improves on past work by resolving the ambiguity caused by multiple acceleration peaks, more accurately identifying the time alignment of fall stages and thus extracting features from the fall stages. Since each stage has a different characteristic, accurate alignment of fall stages significantly improves the fall detection performance in terms of F-score and precision.

The experimental results show that using EvenT-ML yields a significantly better precision and F-score than using either FOSW or FNSW, while still maintaining relatively good recall. As an additional advantage, using EvenT-ML can cost significantly less computationally than using FNSW (with an up to 8-fold run-time reduction) or FOSW (with an up to 78-fold run-time reduction). Compared to existing fall detection approaches (the cascade-classifier approach (CCA), and IMPACT+POSTURE), EvenT-ML achieves a significantly better F-score.

The computational cost, and thus the energy use of the resulting fall detector, can be further reduced by feature selection that considers such cost factors, and this will be examined in future work. Also, some data from additional sensors can be fused to acceleration data, with the aim of increasing the detection rate. Several devices have been shown to give a better result when combined with accelerometer sensors (e.g., Microsoft Kinect [43], gyroscopes [18,44], or barometric altimeters [44]). These type of sensors will be considered for future work. However, we believe that this work is already a step forward towards low-power, non-intrusive, and high-performance fall detection systems.

## Figures and Tables

**Table 1:** Improvements in functionality of the event-triggered machine learning (EvenT-ML) approach compared to Ojetola’s study [18] and the cascade-classifier approach (CCA) [14].

Function	Ojetola’s Study	CCA	EvenT-ML
Feature extraction	Implemented on every segment.	Implemented on segments with a peak higher than a certain threshold.	Implemented on segments with the highest peak during a certain period of time.
Multi-peak detection	N/A	No	Yes

**Table 2:** Types of falls and ADLs (together with their numbers) in the Cogent dataset. ADLs: activities of daily living.

Category	Activity	Number of Events
ADLs	Standing while doing some other activities (e.g., making a phone call)	184
	sitting on a chair while doing some other activities (e.g., reading a book),	184
	near fall,	276
	sitting on the floor (not a result of falling),	276
	lying on a bed while doing some other activities (e.g., reading a book),	92
	walking while doing some other activities (e.g., making a phone call),	184
	unspecified activity	431
Falls	forward (ff)	184
	backward (fb),	93
	left-side (fl),	91
	right-side (fr),	92
	blinded-forward (bff)	92
	blinded-backward (bfb).	92

**Table 3:** Types of falls and ADLs (together with their numbers) in the SisFall dataset.

Category	Activity	Number of Events
ADLs	Walking slowly (D01),	21
	Walking quickly (D02),	21
	Jogging slowly (D03),	21
	Jogging quickly (D04),	21
	Walking upstairs and downstairs slowly (D05),	105
	Walking upstairs and downstairs quickly (D06),	105
	Slowly sitting in a half-height chair, waiting a moment, and standing up slowly (D76),	105
	Quickly sitting in a half-height chair, waiting a moment, and standing up quickly (D08),	105
	Slowly sitting in a low-height chair, waiting a moment, and standing up slowly (D09),	105
	Quickly sitting in a low-height chair, waiting a moment, and standing up quickly (D10),	105
	Sitting a moment, trying to get up, and collapsing into a chair (D11),	105
	Sitting a moment, lying slowly, waiting a moment, and sitting again (D12),	105
	Sitting a moment, lying quickly, waiting a moment, and sitting again (D13),	105
	Being on one’s back, changing to lateral position, waiting a moment, and changing to one’s back (D14),	105
	Standing, slowly bending at the knees, and getting up (D15),	105
	Standing, slowly bending without bending knees, and getting up (D16),	105
	Standing, getting into a car, remaining seated, and getting out of the car (D17),	105
	Stumbling while walking (D18),	105
	Gently jumping without falling, while trying to reach a high object (D19).	105
Falls	Fall forward while walking, caused by a slip (F01),	105
	Fall backward while walking, caused by a slip (F02),	105
	Lateral fall while walking, caused by a slip (F03),	105
	Fall forward while walking, caused by a trip (F04),	105
	Fall forward while jogging, caused by a trip (F05),	105
	Vertical fall while walking, caused by fainting (F06),	105
	Fall while walking, with use of hands on a table to dampen fall, caused by fainting (F07),	105
	Fall forward when trying to get up (F08),	105
	Lateral fall when trying to get up (F09),	105
	Fall forward when trying to sit down (F10),	105
	Fall backward when trying to sit down (F11),	105
	Lateral fall when trying to sit down (F12),	105
	Fall forward while sitting, caused by fainting or falling asleep (F13),	105
	Fall backward while sitting, caused by fainting or falling asleep (F14),	105
	Lateral fall while sitting, caused by fainting or falling asleep (F15).	105

**Table 4:** F-scores (average and standard deviation) of *k*-nearest neighbor (*k*-NN), logistic regression (LR), and support vector machine (SVM) when $t_{mp}=1$ s, $t_{sg}=1$ s and τ=1.8 g are used on EvenT-ML. RBF: radial basis function.

Metrics	*k*-NN (%)			LR (%)			SVM (%)		
	k=1	k=2	k=3	$C=10^{8}$	$C=10^{9}$	$C=10^{10}$	Linear	RBF	Polynomial
F-score	92.7 ± 7.9	91.4 ± 11	94 ± 8.8	97.5 ± 3.3	97.6 ± 3.3	97.5 ± 3.2	95.7 ± 8.2	93.5 ± 10.3	94.6 ± 10.3

**Table:** (**a**)

Approach	CART		*k*-NN		LR		SVM	
	Cogent	SisFall	Cogent	SisFall	Cogent	SisFall	Cogent	SisFall
EvenT-ML	91.4 ± 8.8	83.5 ± 4.8	95.6 ± 7.2	87.5 ± 5.1	97.2 ± 4.1	88.4 ± 5.1	97.2 ± 5.6	90.3 ± 5
CCA	86.6 ± 12.3	82.9 ± 3.9	83.1 ± 11.5	81.1 ± 4.2	89.6 ± 6.9	86.3 ± 5	87.2 ± 8.3	83.1 ± 4.9
FNSW	44.5 ± 9.4	52.3 ± 1.3	72.6 ± 12.6	53.5 ± 2.1	91.7 ± 11.3	51.9 ± 1.7	94.5 ± 8.4	50.7 ± 0.6
25%-FOSW	41.2 ± 8.2	51.1 ± 1.3	66.3 ± 13.3	52.3 ± 2.1	90.1 ± 11.6	51.4 ± 1.7	93.2 ± 9.5	50.3 ± 0.5
50%-FOSW	35.3 ± 6.4	49.5 ± 0.6	59.3 ± 12.7	50.2 ± 0.9	89.8 ± 11.4	50.6 ± 1.4	93.4 ± 9.8	49.9 ± 0.4
75%-FOSW	27.8 ± 4.3	48.9 ± 0.3	44.7 ± 9.3	49.2 ± 0.5	88.6 ± 11.4	50 ± 0.8	93.1 ± 10.2	49.7 ± 0.3
90%-FOSW	21.4 ± 2.4	48.7 ± 0	29.4 ± 5.4	48.8 ± 0.1	87.5 ± 12.3	49.6 ± 0.3	92.5 ± 10.8	49.6 ± 0.2

**Table:** (**b**)

Approach	CART		*k*-NN		LR		SVM	
	Cogent	SisFall	Cogent	SisFall	Cogent	SisFall	Cogent	SisFall
EvenT-ML	92.4 ± 11.2	92.5 ± 7.7	93.2 ± 12.1	94.5 ± 5.8	98.1 ± 3.8	94.6 ± 4.8	94.7 ± 11	92.7 ± 8.9
CCA	84.6 ± 14.9	84.6 ± 11.3	88.8 ± 13.5	87.7 ± 9.5	89.3 ± 15.6	83.8 ± 13.8	88.5 ± 15.9	86.1 ± 14.7
FNSW	92.4 ± 10.1	99.8 ± 0.9	89.9 ± 13.9	99.9 ± 0.3	87.3 ± 17.9	99.9 ± 0.3	83.9 ± 20.7	100 ± 0
25%-FOSW	94.6 ± 7.7	99.9 ± 0.3	91.1 ± 12.8	100 ± 0	90.4 ± 15.4	100 ± 0	85.6 ± 20.2	100 ± 0
50%-FOSW	97 ± 4.9	100 ± 0	95.2 ± 8.8	100 ± 0	92.9 ± 13.1	100 ± 0	85.7 ± 20.1	100 ± 0
75%-FOSW	98.8 ± 3.8	100 ± 0	98.1 ± 5.3	100 ± 0	94.4 ± 10.8	100 ± 0	86.3 ± 20	100 ± 0
90%-FOSW	99.7 ± 1.5	100 ± 0	99.7 ± 1.5	100 ± 0	95.2 ± 10.7	100 ± 0	86.6 ± 19.8	100 ± 0

**Table:** (**c**)

Approach	CART		*k*-NN		LR		SVM	
	Cogent	SisFall	Cogent	SisFall	Cogent	SisFall	Cogent	SisFall
EvenT-ML	91.6 ± 9.3	87.7 ± 5.5	94 ± 8.8	90.7 ± 4.2	97.6 ± 3.3	91.3 ± 3.3	95.7 ± 8.2	91.1 ± 5.2
CCA	83.9 ± 7.1	83.3 ± 7.5	84.5 ± 6.5	84.1 ± 6.4	84.6 ± 9.8	84.4 ± 10	84 ± 10.3	83.8 ± 10.2
FNSW	59.6 ± 9.5	68.6 ± 1.1	79.7 ± 11.5	69.7 ± 1.8	88.3 ± 13.3	68.3 ± 1.4	87.2 ± 15.7	67.3 ± 0.5
25%-FOSW	56.9 ± 8.2	67.6 ± 1.1	76.1 ± 11.8	68.6 ± 1.8	89.2 ± 11.6	67.9 ± 1.5	87.9 ± 15.5	66.9 ± 0.5
50%-FOSW	51.5 ± 7.1	66.2 ± 0.5	72.5 ± 10.7	66.8 ± 0.8	90.6 ± 10.4	67.2 ± 1.2	87.9 ± 15.3	66.5 ± 0.3
75%-FOSW	43.2 ± 5.3	65.7 ± 0.3	60.9 ± 9.5	66 ± 0.4	90.8 ± 9	66.7 ± 0.7	88.2 ± 15.5	66.4 ± 0.3
90%-FOSW	35.2 ± 3.2	65.5 ± 0	45.2 ± 6.4	65.6 ± 0.1	90.5 ± 9.3	66.3 ± 0.3	88 ± 15.24	66.3 ± 0.2

**Table 6:** Average and standard deviation of segments and their total computational cost for each segmentation technique on a subject.

Approach	Number of Segments Generated		Total Computational Cost for Each Subject (ms)	
	Cogent	SisFall	Cogent	SisFall
EvenT-ML	38.4 ± 9.9	323.7 ± 38.7	34.3 ± 8.7	473.5 ± 53.7
FNSW	429.4 ± 84.8	873 ± 0	269.5 ± 54.6	1000 ± 35
FOSW 25%	572.3 ± 113.1	1163.9 ± 0.3	367.3 ± 79.9	1300 ± 48
FOSW 50%	858.2 ± 169.6	1745 ± 0	555.2 ± 113.1	2100 ± 51
FOSW 75%	1715.8 ± 339.2	3489.9 ± 0.3	1098.9 ± 219.8	3800 ± 25
FOSW 90%	4288.8 ± 848.4	8723.8 ± 0.7	2528.3 ± 498	94,500 ± 52

**Table 7:** IMPACT+POSTURE performance (average and standard deviation) on Cogent and SisFall datasets.

Datasets	Precision (%)	Recall (%)	F-score (%)
Cogent	90.9 ± 10.2	87.6 ± 11.9	88.6 ± 9.2
SisFall	54.1 ± 1.7	100 ± 0	70.2 ± 1.4
